# A chromosome-level genome assembly of *Sesamia inferens*

**DOI:** 10.1038/s41597-024-02937-6

**Published:** 2024-01-25

**Authors:** Hongran Li, Yan Peng, Chao Wu, Chess-Kadouste Vigan, Kaikai Mao, Jingyun Zhu, Luming Zou, Minghui Jin, Lei Zhang, Yutao Xiao

**Affiliations:** Shenzhen Branch, Guangdong Laboratory of Lingnan Modern Agriculture, Key Laboratory of Gene Editing Technologies (Hainan), Ministry of Agriculture and Rural Affairs, Agricultural Genomics Institute at Shenzhen, Chinese Academy of Agricultural Sciences, Shenzhen, 518116 P. R. China

**Keywords:** Genomics, Evolutionary genetics

## Abstract

The pink stem borer, *Sesamia inferens* (Walker), is a significant polyphagous pest historically restricted to regions south of N34° latitude. However, with changes in global climate and farming practices, the distribution of this moth has progressively exceeded its traditional limit of 34° N and encompassed most regions in North China. The genetic adaptations of *S. inferens* remain incompletely understood due to the lack of high-quality genome resources. Here, we sequenced the genome of *S. inferens* using PacBio and Hi-C technology, yielding a genome assembly of 865.04 Mb with contig N50 of 1.23 Mb. BUSCO analysis demonstrated this genome assembly has a high-level completeness of 96.1% gene coverage. In total, 459.72 Mb repeat sequences (53.14% of the assembled genome) and 20858 protein-coding genes were identified. We used the Hi-C technique to anchor 1135 contigs to 31 chromosomes, yielding a chromosome-level genome assembly with a scaffold N50 of 29.99 Mb. In conclusion, our high-quality genome assembly provided valuable resource that exploring the genetic characteristics of local adaptation and developing an efficient control strategy.

## Background & Summary

The pink stem borer, *Sesamia inferens* (Walker) (Lepidoptera: Noctuidae), is a significant polyphagous pest that damages a wide range of food crops including rice, maize, sorghum, and barley^1,2^. In recent decades, the impact of *S. inferens* has been steadily rising in most areas along the Yangtze River and coastal regions in southern China. In certain locations, the damage caused by this moth has even surpassed that of the *Chilo suppressalis*, propelling it from a secondary pest to a primary and significant threat^3,4^. The *S. inferens* populations likely have an abundance of ecological phenotypes to adapt to complex environmental conditions. For example, the number of generations per year decreases with increasing latitude, which is largely affected by the length of the growing season^5^. It consumes internal tissues of sugarcane, and the distinctive “deadheart” symptom manifests when it feeds on young plants. Infestation in older plants may lead to a decrease in growth, and stems may exhibit desiccation^6^. Furthermore, there have been studies concentrating on the characterization of insecticide resistance in *S. inferens*. For instance, a population resistant to Fipronil exhibited a 106.00- and 22.71-fold resistance compared to unselected and field strains^7,8^ documented the susceptibility of three field populations of *S. inferens* to chlorantraniliprole and flubendiamide. They also conducted the cloning and characterization of the full-length cDNA of the ryanodine receptor, along with profiling its mRNA expression pattern.

The *S. inferens* is widely distributed in Asian countries such as China, Japan, India, Laos and Pakistan^9–13^, and was recently discovered in Hawaii and Guam (United States) according to Centre for Agriculture and Bioscience International (CABI) (https://www.cabi.org/isc/datasheet/49751#REFDDB-202162). In China, the natural range of this species was traditionally confined to areas south of N34° latitude^14^. The *S. inferens* exhibited limited ability for migratory flight, as 75.5% of the moths had a cumulative flight distance of ≤5.0 km^15^. Thus, during winter, the *S. inferens* predominantly overwinters as mature larvae on the roots of rice, water bamboo, and various weed species^4^. However, due to the change of global climate and farming system, the distribution of the moth has progressively exceeded its traditional limit of 34° N and expanded to include the majority of regions in North China^4,16^. Given the overwintering ability being one of the main factors restricting the distribution of the moth, it is particularly important to study the adaptable ability of the overwintering larvae of the moth in North China.

While the severe damage to rice and other crops caused by *S. inferens* has gained significant attention, the current efforts primarily focus on the field dynamics and forecast, environmental adaption, biological control and pesticides resistance assessment^7,8,10,17–20^. Indeed, the knowledge of the population structure and genetic bases of the rapid adaptation of *S. inferens* in its expanded habitat are limited. Only a few studies have been conducted to explore the genetic diversity of *S. inferens*, using a smaller number of molecular markers^21,22^. These findings were based on mitochondrial DNA or microsatellite markers, which either reflected the maternal history or were limited by a lack of sufficient genetic information. Thus, the genetic adaptations of *S. inferens* in North China remain to be confirmed using whole genomic variation. Yet, the lack of a high-quality reference genome seriously limits understanding the extent to which genetic variation resulted in *S. inferens’s* expanding range and adaptations to local climactic regimes.

Here, we generated a chromosome-level genome assembly of *S. inferens* using PacBio and Hi-C technology. Phylogenetic analysis was performed to determine the relationship of *S. inferens* with other Noctuidae species. Moreover, functional enrichment showed that the rapidly expanded gene families and positively selected genes were associated with multiple metabolism-related pathways that contributed to the local adaptation in new environment. Our study provides the first genome assembly for the pink stem borer, which will facilitate studies on the genetic mechanisms of evolutionary adaptation for in large-scale habitats and agro-ecosystems across China and also significantly benefit efforts to control this important rice pest.

## Methods

### Sample collection and genomic sequencing

The pink borer samples were collected from Guangdong Province, China in 2022 and were reared under the controlled conditions (27 ± 2 °C, 16 L: 8D and RH 60 ± 5%) in the laboratory for one generation to obtain sibling pupa for Hi-C and genome sequencing. To effectively eliminate microbial contaminants present on the surface of the pupa, the pupa sample underwent surface sterilization through a triple treatment process involving 75% alcohol and sodium hypochlorite solution.

For PacBio sequencing, genomic DNA was extracted from one male pupa and then subjected to sequencing on the Pacific Biosciences Sequel II platform to generate HiFi reads in circular consensus sequencing (CCS) mode. A total of 24 Gb (~29.26 × coverage) single molecule real-time (SMRT) long reads were generated. In parallel, Genomic DNA was extracted from another pupa of the same parent to facilitate the construction of Hi-C libraries. These libraries were generated using the MboI restriction enzyme and subsequently subjected to sequencing using the Illumina Novaseq/MGI-2000 platform. This phase of the study produced ~86.9 Gb (~100 × coverage) of data with 150 bp paired-end sequencing raw reads.

### RNA sequencing

For the purpose of genome annotation in *S. inferens*, total RNA was isolated from larvae, pupae, and adults employing the TRIzol reagent from Invitrogen, USA. Subsequently, a cDNA library was generated utilizing the NEBNext Ultra RNA Library Prep Kit designed for Illumina (NEB), in accordance with the provided instructions. RNA-seq libraries were constructed and sequenced using Illumina HiSeq X Ten (insert size 240 bp,150 PE reads) at Novogene, Tianjing. As a result, a total of 61.27 Gb sequencing data was generated.

### Genome assembly

The estimation of genome size relied on analyzing the distribution of k-mer frequencies. Here, 21-kmer analysis of Illumina paired-end sequencing reads was performed using Jellyfish v2.1.3 software^23^, while GenomeScope 2.0^24^ was employed for calculating heterozygosity. As a result, the genome size of *S. inferens* was projected to be approximately 865.04 Mb (Fig. S1).

To eliminate CCS reads containing residual PacBio adapter sequences, the HiFiAdapterFilt software was applied^25^. The ensuing clean PacBio reads were then assembled utilizing default settings in Hicanu v2.0^26^. The software purge_dups was subsequently utilized for the removal of haplotigs and contig overlaps^27^. To gauge the assembly’s comprehensiveness, Benchmarking Universal Single-Copy Orthologs v5.1.2 (BUSCO) was employed, using homologous genes from insecta_odb10. The outcomes exhibited a successful detection rate of 96.1% for BUSCO genes, of which 94.7% were identified as complete and single-copy genes and 1.4% were categorized as complete and duplicated (Table 1). Following these evaluations, a total of 1135 contigs were assembled, with a contig N50 size measuring 1.23 Mb (Table 2).

**Table 1:** Assessment of completeness of the *S. inferens* assembly.

Category	Number of BUSCOs
C: 96.1% [S:94.7%, D:1.4%], F: 0.9%, M: 3.0%	1658
Complete BUSCOs (C)	1593
Complete and single-copy BUSCOs (S)	1570
Complete and duplicated BUSCOs (D)	23
Fragmented BUSCOs (F)	15
Missing BUSCOs (M)	50

**Table 2:** Comparison of genome assemblies in six Noctuidae species.

	*S. inferens*	*A. ipsilon*	*S. frugiperda*	*S. litura*	*H. armigera*	*T. ni*
Genome size (Mb)	865.04	515	390.38	438.32	356.67	333.0
Karyotype	—	—	30 + Z	30 + Z	30 + Z	30 + Z
Number of contigs	1135	274	776	13636	106	7885
Number of scaffolds	69	31	—	3597	41	1916
Max scaffold length (Mb)	38.00	—	21.4	4.4	17.88	8.92
Contig N50 (kb)	1231.9	6747	5606.9	68.4	7516.16	140.0
Scaffold N50 (Mb)	28.60	17.57	12.69	13.44	12.27	4.51
BUSCO (%)	96.1	98.1	—	99.0	97.7	98.8
G + C (%)	38.62	38	36.4	36.6	36.53	35.5
Repeat (%)	53.14	32.09	27.2	31.8	27.04	16.7
Number of genes	18937	19412	22260	15317	18668	14384

Note: *A. ipsilon*^43^, *S. frugiperda*^44^, *S. litura*^45^, *H. armigera*^46^, *T. ni*^47^.

### Hi-C scaffolding

**Fig. 1:**
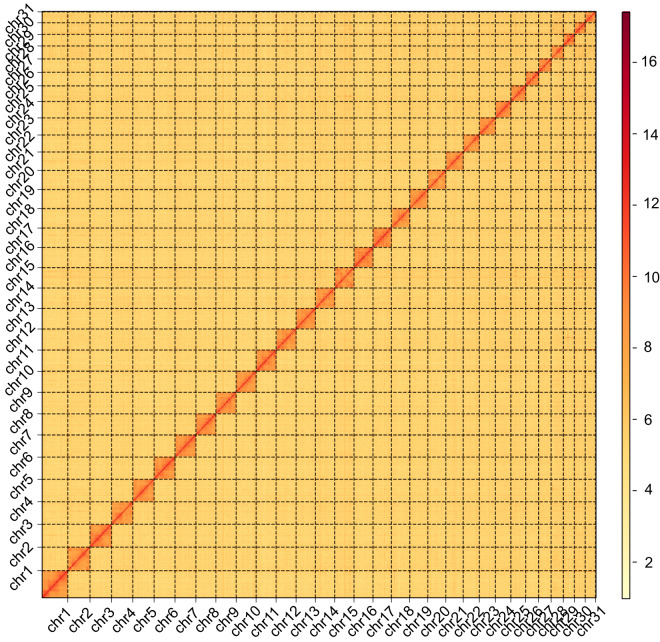
Heatmap of genome-wide Hi-C data of the pink borer, *Sesamia inferens*. Linkage group contact map informed by Hi-C sequencing data in the genome. Thirty-one linkage groups were generated after the clustering of contact map. The colour bar indicates the frequency of Hi-C interaction intensity from low (yellow) to high (red) in the plot.

Initial processing involved the removal of low-quality raw reads (those with a quality score <20 and shorter than 30 bp), along with adapter sequences, through the utilization of FASTP v0.20.0. Subsequently, the purified reads were aligned to the contig assembly using BOWTIE2 v2.3.2 with specific parameters (-end-to-end–very-sensitive -L 30)^28^. The Hi-C reads were used to map the draft genome using Juicer v1.5 and corrections for misjoins, ordering, and orientation were carried out using 3D-DNA v.180922 with the parameter (-r 0)^29,30^. As a results, Hi-C data were combined with the contig-level assembly to generate a chromosome-level assemble. These contigs were improved to generate 69 scaffolds (865.04 Mb) with a scaffold N50 size of 29.99 Mb (Table 2; Table S1). Finally, Hi-C data were employed for the anchoring, ordering, and orientation of these scaffolds, yielding 31 chromosomes (Chr1-Chr31), harboring >99.05% of assembled sequences (Fig. 1).

### Repeat annotation

**Fig. 2:**
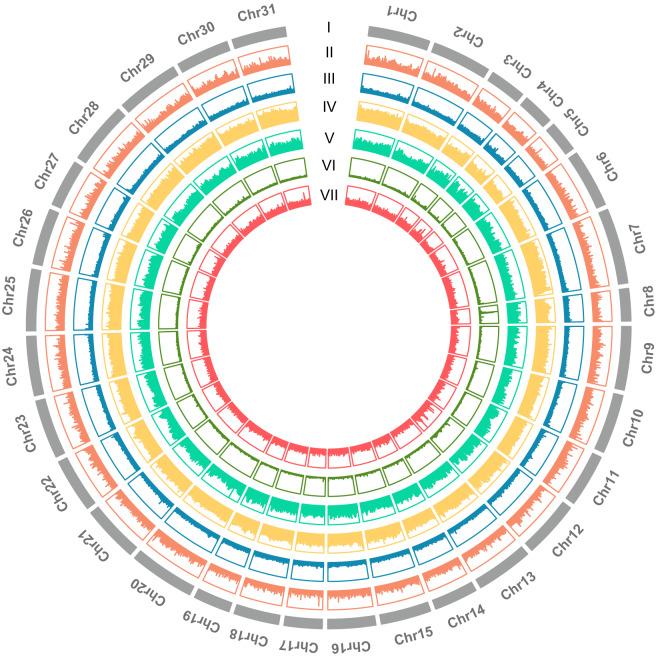
Circular representation of the chromosomes of the pink borer, *Sesamia inferens*. The genome information landscape map, from the outer ring, I: chromosome ideograms (Mb scale), II: Density of protein-coding genes; III: Density of G + C content; IV: Density of genome-wide single nucleotide polymorphism (SNP) sites; V: Density of long interspersed nuclear elements (LINEs); VI: Density of long terminal repeats (LTR); VII: Density of DNA transposons.

We customized a de novo repeat library of the genome using RepeatModeler open-1.0.11. RepeatMasker v2.1 (http://www.repeatmasker.org/)^31^ was used to compare the genome sequence with the known repeat sequences in the reference database (Repbase). The Tandem Repeats Finder (TRF) package v4.09 was used to identify tandem repeat sequences in the *S. inferens* genomes^32^. In total, 459.72 Mb sequences (approximately 53.14% of the assembled genome) were identified as repeat sequences (Fig. 2). Among them, long interspersed nuclear elements (LINE) (18.01%), DNA transposons (5.12%), and short interspersed nuclear elements (SINE) (5.00%) represented the top three most abundant repeat types. Unknown repeat types accounted for 15.67% of identified repeat regions (Table S2).

### Gene prediction and functional annotation

Three strategies including ab initio prediction, homology search and transcriptome-based approaches were integrated predict protein-coding genes. For homology-based annotation analysis, the genome sequences of *S. inferens* were cross-referenced with the protein-coding sequences of related species (*Helicoverpa armigera, Manduca sexta, Spodoptera litura, Trichoplusia ni*) through BLAST^33^ and GeneWise v2.4.1^34^ to deduce gene structures. For transcriptome-based prediction, HISAT v2.147^35^ was used to align the transcriptome data to the genome, and gene information was predicted using StringTie v1.3.4c^36^. For the ab initio method, the software packages Augustus v3.2.2^37^ was employed with default settings. The consolidated gene models from the ab initio, homology-based, and RNA-seq-based methods were then integrated using EvidenceModeler v1.1.0 with default settings^38^ to yield a comprehensive gene dataset. A total of 20,858 protein-coding genes within the *S. inferens* genome were generated through the merging of these approaches (Table S3).

Subsequently, the functional annotation of the protein-coding genes was performed against databases like the non-redundant protein database (NR), Kyoto Encyclopedia of Genes and Genomes (KEGG), eggNOG, and Trembl using BLASTP v2.7.1 with a threshold of 1e-5. This analysis revealed that functional annotations were possible for 18,937 genes, equating to 90.79% of the total (Table S3).

## Data Records

The genome assembly utilized PacBio and Hi-C sequencing techniques, and the data have been stored in the NCBI Sequence Read Archive with the accession numbers SRR25638298 and SRR25638299 under accession number SRP455073^39^. Additionally, the transcriptome data used for genome annotation are also available in the NCBI Sequence Read Archive with accession numbers ranging from SRR25638295 to SRR25638297 (The detailed information of the raw data in Table S4). The final genome assembly and annotation data have been deposited in the figshare database (10.6084/m9.figshare.23036267.v1)^40^. The genome assembly has been also deposited at GenBank under the accession GCA_035079275.1^41^.

## Technical Validation

Three validation methods were employed to assess the contiguity, accuracy and completeness of the genome assembly. Firstly, a total of 1135 contigs were assembled, with a contig N50 size measuring 1.23 Mb. We then used the Hi-C data combined with the contig-level assembly to generate a chromosome-level assemble that spanned 865.04 Mb, characterized by a scaffold N50 value of 29.99 Mb. Secondly, the Hi-C heatmap unveiled a discernible and well-structured pattern of interaction contacts along the diagonals within and around the chromosome inversion region, resulted in the encapsulation of over 99.05% of the assembled sequences within these chromosomes (Fig. 1). Finally, we employed Benchmarking Universal Single-Copy Orthologs (BUSCOv5.1.2), using homologous genes from insecta_odb10. The results indicated that 96.1% of BUSCO genes (insecta_odb10) were successfully detected within the genome assembly (Table 1). Among them, 94.7% were identified as single-copy genes and 1.4% were categorized as duplicated. These observations indirectly validated the precision and accuracy of the chromosome assembly.

To ensure the completeness and accuracy of the annotated gene set, the forecasted gene models underwent a comparison with multiple protein databases including nr, eggNOG, Trembl, and KEGG. The outcome demonstrated that 18,937 (90.79%) of the projected gene models exhibited notable homology with proteins found within at least one of these databases. Moreover, clean reads from three transcriptomes of larvae, pupa and adults were mapped onto the genome assembly, more than 90.5% of the RNA-Seq reads can be aligned to the coding regions of the reference genome.

### Supplementary information

Supplementary material for SciData

## Data Availability

All software and pipeline were executed following the instructions and protocols provided by the respective bioinformatic tools’ publications. All the scripts for the genome assemble we have deposited in a public repository 10.6084/m9.figshare.24570898.v1^42^. The software versions and corresponding code/parameters used are comprehensively outlined in the Methods section.
