# Supplementary material for: A chromosome-level genome assembly of Sesamia inferens
Source: Sci Data. 2024 Jan 25;11:134. doi: 10.1038/s41597-024-02937-6 (PMC10810861; doi:10.1038/s41597-024-02937-6)
Supplement: Supplementary file 1 — Supplementary material for SciData [file 41597_2024_2937_MOESM1_ESM.docx]

**A chromosome-level genome assembly of *Sesamia inferens***

Hongran Li^#^, Yan Peng^#^, Chao Wu^#^, VIGAN Chess-Kadouste^#^, Kaikai Mao, Jingyun Zhu, Luming Zou, Minghui Jin, Lei Zhang, Yutao Xiao^1*^

Shenzhen Branch, Guangdong Laboratory of Lingnan Modern Agriculture, Key Laboratory of Gene Editing Technologies (Hainan), Ministry of Agriculture and Rural Affairs, Agricultural Genomics Institute at Shenzhen, Chinese Academy of Agricultural Sciences, Shenzhen, 518116, P. R. China

^#^These authors contributed equally to this work.

^*^To whom correspondence should be addressed: Yutao Xiao (xiaoyutao@caas.cn).


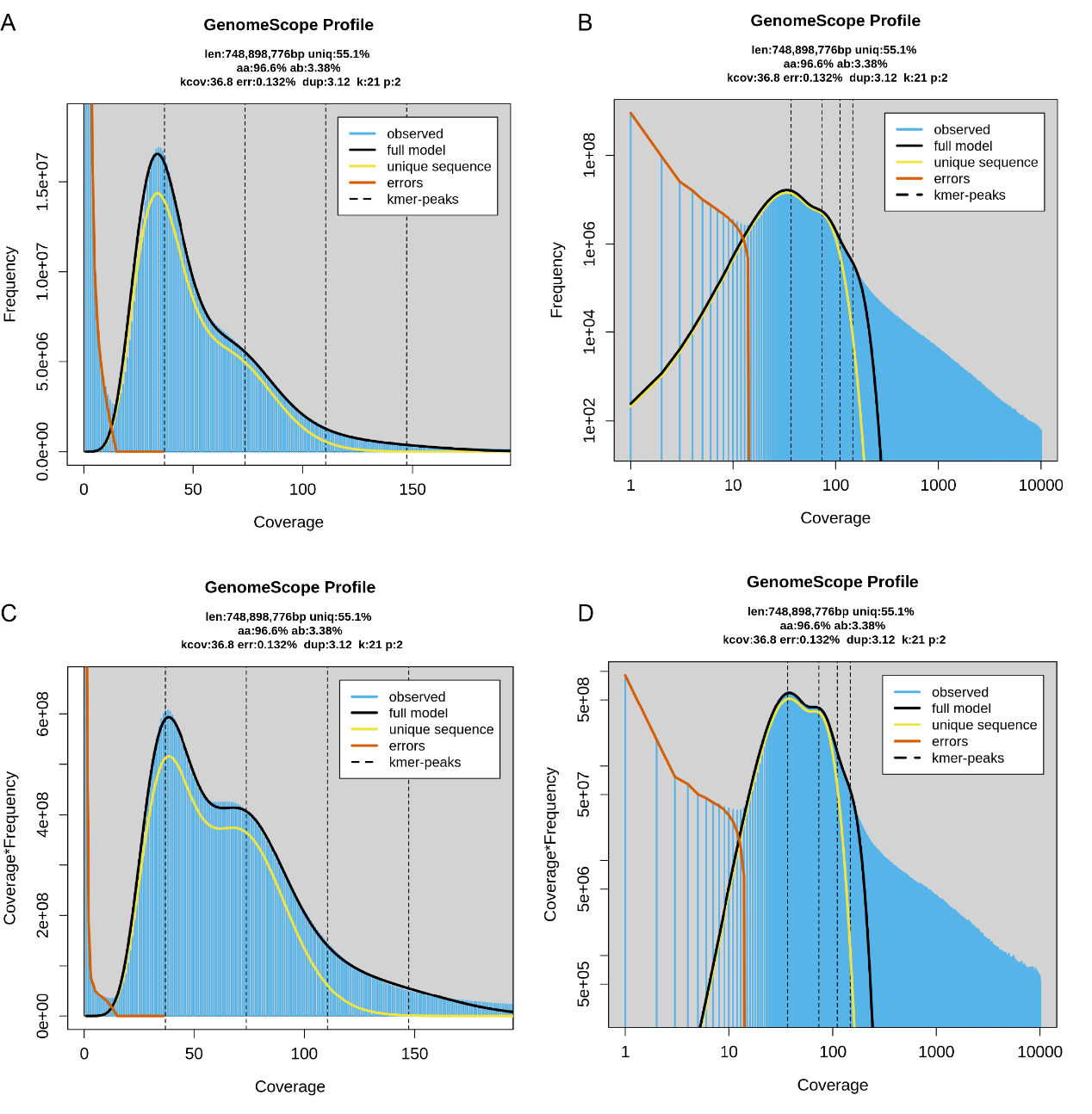


**Fig. S1 Genome survey result based on k-mer frequency analysis. K-mer frequency analysis was performed using Jellyfish (k-mer = 21) based on Illumina paired-end sequencing reads of genomic DNA.** Genome size, repeat sequence content, and heterozygosity ratio were estimated based on k-mer frequency distribution using GenomeScope 2.0. The estimated genome size was 748.89 Mb.

Table S1 Summary statistics of genome assembly of *Sesamia inferens*

| Item | Scaffold number | Scaffold length |
| --- | --- | --- |
| Total Length | 1 | 865044522 |
| Total N Length | 545081 | 0.000629722 |
| Maximum Length | 1 | 39855675 |
| Minimum Length | 1 | 25000 |
| Average Length | - | 12536900 |
| Median Length | - | 271255 |
| N10 Length | 3 | 33498179 |
| N20 Length | 5 | 32992099 |
| N30 Length | 8 | 31317297 |
| N40 Length | 11 | 31001768 |
| N50 Length | 14 | 29986663 |
| N60 Length | 17 | 28575689 |
| N70 Length | 20 | 28013015 |
| N80 Length | 23 | 24984587 |
| N90 Length | 27 | 19422890 |

Table S2 Statistics of repeat elements of *Sesamia inferens*

| Repeat type | Number of elements | Length occupied (bp) | Percentage of sequence |
| --- | --- | --- | --- |
| DNA | 130023 | 44303513 | 5.12 |
| LINE | 484861 | 155759458 | 18.01 |
| SINE | 205095 | 43229393 | 5.00 |
| LTR | 60523 | 31555664 | 3.65 |
| Simple repeat | 121757 | 12146611 | 1.40 |
| Satellite | 880 | 373446 bp | 0.04 |
| unknown | 607638 | 135536991 | 15.67 |
| Total | 1788617 | 459721605 | 53.14 |

Table S3 Summary statistic of gene functional annotation in *Sesamia inferens* genome

| Item | Annotated genes | Percentage |
| --- | --- | --- |
| kegg | 14818 | 71.0423% |
| Nr | 18686 | 89.5867% |
| eggNOG | 18805 | 90.1573% |
| Trembl | 18540 | 88.8868% |
| total | 18937 | 90.7901% |

Table S4 The detailed information of the upload raw data at NCBI in our study

| Title | Description | Accession | BioSample | Release Date | SRA. filename |
| --- | --- | --- | --- | --- | --- |
| szd1 | HiC data | SRR25638298 | SAMN36950133 | 2023/8/18 | SZD1_S24_L001_R1_001.fastq.gz, SZD1_S24_L001_R2_001.fastq.gz |
| szd2 | Hifi data | SRR25638299 | SAMN36950132 | 2023/8/18 | SZD2.hifi.fastq.gz |
| szl | raw data of Larvae transcriptome | SRR25638296 | SAMN36950135 | 2023/8/18 | SZL_1.clean.fq.gz,  SZL_2.clean.fq.gz |
| szp | raw data of pupal transcriptome | SRR25638295 | SAMN36950136 | 2023/8/18 | SZP_1.clean.fq.gz, SZP_2.clean.fq.gz |
| sza | raw data of adut transcriptome | SRR25638297 | SAMN36950134 | 2023/8/18 | SZA_1.clean.fq.gz, SZA_2.clean.fq.gz |
